# Epidemiological, Clinical, and Evolutionary Profile of Helicobacter pylori Infection in the Pediatric Population of the Eastern Region of Morocco: A Series of 118 Cases

**DOI:** 10.7759/cureus.79449

**Published:** 2025-02-22

**Authors:** Maria Rkain, Hanae Bahari, Amal Hamami, Aziza Elouali, Abdeladim Babakhouya

**Affiliations:** 1 Department of Pediatrics, Mohammed VI University Hospital, Oujda, MAR; 2 Faculty of Medicine and Pharmacy, Mohammed First University, Oujda, MAR

**Keywords:** children, clinical manifestation, gastritis, helicobacter pylori, proton-pump inhibitors

## Abstract

Introduction:* Helicobacter pylori* *(H. pylori)* is a bacterium that affects a significant portion of the global population and can lead to gastroduodenal ulcers and gastric cancers in adulthood. In pediatric practice, *H. pylori* infection is a common concern, although most affected children remain asymptomatic. This study aims to describe the epidemiological, clinical, endoscopic, and histological profile of *H. pylori *gastritis in the pediatric population of the Eastern region of Morocco.

Materials and methods: Patients aged between one and 16 years who underwent upper gastrointestinal endoscopy between January 2022 and June 2024 were included in this study. Gastric biopsies were taken, and the presence of *H. pylori *infection was confirmed by Giemsa staining. Demographic data and clinical and endoscopic characteristics were collected, along with histopathological results according to theSydneysystem.

Results: Among the 230 children studied, 118 (51%) were infected with *H. pylori*, with the prevalence of infection increasing with age, notably in children aged between 10 to 16 years (46.61%). A female predominance was observed, representing 59% of the cases. The majority of children (68%) came from disadvantaged socioeconomic backgrounds. Abdominal pain was the primary symptom, reported in 60.5% of infected children. All patients exhibited macroscopic gastritis, with petechial and erosive features found in 59% and 62% of cases, respectively. Histologically, *H. pylori *gastritis was active in 87.2% of cases in the antrum, with a follicular pattern observed in 43.2%. Gastric atrophy was present in 25.42% of the children. The *H. pylori* eradication rate in our study was 94.92%, with therapeutic failure observed in 5.08% of patients, mainly due to insufficient treatment adherence.

Conclusion:* Helicobacter pylori* infection can cause several gastrointestinal issues, and early detection and treatment are important to prevent complications and promote successful eradication.

## Introduction

*Helicobacter pylori* (*H. pylori*) is a Gram-negative, microaerophilic bacterium that typically colonizes the gastric mucosa during childhood. This bacterium is associated with the development of chronic active gastritis, peptic ulcer disease, gastric cancer, and mucosa-associated lymphoid tissue lymphoma later on during adulthood [1]. While the exact transmission pathways remain partially understood, infection is primarily thought to occur through direct human-to-human contact or environmental contamination [2]. Factors such as the number of siblings, parental education levels, water sources, and waste management practices have been identified as significant risk factors for *H. pylori* infection in the pediatric population [3].

In children, the severity of *H. pylori* infection is typically lower compared to adults, with a virtually nonexistent incidence of gastric malignancies. The infection is often asymptomatic in this age group. Research has shown no significant correlation between *H. pylori* infection and abdominal pain or other abdominal symptoms in children [4].

There are several diagnostic methods for *H. pylori* infection, some of which are invasive and require gastric biopsy, while others are non-invasive [5]. This study aims to describe the epidemiological, clinical, endoscopic, and histological profile of *H. pylori *gastritis in the pediatric population of the Eastern Region in Morocco.

## Materials and methods

A retrospective study was conducted at the Mohammed VI University Hospital in Oujda, Morocco, from January 2022 to June 2024. Patients under the age of 16 who required diagnostic or therapeutic upper digestive endoscopy and had not taken any medication (antibiotics or proton pump inhibitors) in the 30 days prior to the test were randomly included in this study. Informed parental consent was obtained. Endoscopic lesions were captured during the procedure, and biopsy samples were taken from the duodenal, antral, angular, and fundic regions. These samples were fixed in 10% formalin. Histological sections were stained with Giemsa. Histological variables were classified according to the Sydney system's visual analog scale [6]. Patients were considered infected when *H. pylori *was detected in the gastric samples. All *H. pylori*-positive patients received the same standard eradication treatment and completed it. No patient received a different treatment regimen.

Statistical analyses were performed using Microsoft Excel (Microsoft Corp., Redmond, WA) to analyze the data. Descriptive statistics, including mean and frequency distributions, were calculated for all variables.

## Results

Prevalence and demographics

The prevalence of *H. pylori* infection in our study was 51% (118/230). The average age of the patients was nine years, with ages ranging from one to 16 years. The highest prevalence (46.61%) was observed in the age group of 10 to 16 years. The study population consisted of 118 children: 48 boys (41%) and 70 girls (59%), showing a clear female predominance with a sex ratio of 1.45 (Table 1).

**Table 1:** Demographic characteristics of the patients

Variable	Number of cases	Percentage
Sex (male)	48 cases	40.67%
Sex (female)	70 cases	59.32%
Age (Under 5 years )	17 cases	14.40%
Age (5-10 years)	46 cases	38.98%
Age (Over 10 years )	55 cases	46.61%

Geographic and socioeconomic background

The majority of patients were from Oujda, with others coming from neighboring cities in the Eastern region. Among the patients, 68% (n=80) had a low socioeconomic status, which contributed to the high prevalence of the infection; 38.13% of patients (n=45) had iron-deficiency anemia, celiac disease, or type 1 diabetes. Regarding family history, *H. pylori* gastritis was noted in three mothers (2.54%), and four patients had a family history of *H. pylori *gastritis in their siblings (3.38%).

Duration and reasons for consultation

It was observed that more than half of the children (72.03%, n=85) consulted after experiencing symptoms for more than six months, partly explaining the delayed diagnosis. The main reason for consultation was abdominal pain, present in 60.5% of patients (with 77% of cases localized to the epigastric region). The pain was isolated in 24% of cases or associated with other symptoms in 84.6%, notably vomiting, pyrosis, and upper gastrointestinal bleeding. Other clinical signs observed included skin and mucosal pallor and stunted growth.

Endoscopic findings

All patients exhibited macroscopical gastritis, with petechial and erosive lesions being the most frequent, found in 59% and 62% of cases, respectively. Other findings included a nodular appearance in 50% of cases, a congested appearance in 43%, an erythematous-white appearance in 25%, and erythematous and ulcerative-bulbar appearances in 18% and 2% of cases, respectively.

Histopathological findings

The histopathological study was performed on all patients (n=118), enabling the analysis of various histological parameters. *Helicobacter pylori *was detected in the antrum of 70.18% of patients (n=80), with active *H. pylori* gastritis observed in 87.2% (n=103) of cases and a follicular appearance in 43.2% (n=51). Atrophy was identified in 30 patients, accounting for 25.42%. In the fundus, *H. pylori* infection showed an activity rate of 48.3% in 57 patients, with a follicular appearance in 13.55% (n=16), while atrophy was present in 6.77% of cases (n=8) (Table 2).

**Table 2:** Histological findings of the patients

Particulars		Histological appearance	Number of cases	Percentage
Antritis	Atrophy	Mild	24	20.33%
		Moderate	6	5.08%
		Severe	0	0%
	Activity	Mild	77	65.25%
		Moderate	24	20.33%
		Severe	2	1.69%
	Follicular appearance	Mild	27	22.88%
		Moderate	23	19.49%
		Severe	1	0.84%
	*Helicobacter pylori*-positive		80	70.17%
Fundus	Activity	Mild	48	40.67%
		Moderate	9	7.62%
	Atrophy	Mild	6	5.08%
		Moderate	2	1.69%
	Follicular appearance	Mild	16	13.55%
	*Helicobacter pylori*-positive		34	29.82%
Duodenum		Non-specific	71	60.16%

Treatment and follow-up

The treatment was based on the administration of a PPI combined with antibiotics. The antibiotic regimen lasted for 14 days, with amoxicillin used during the first week, followed by clarithromycin and metronidazole in the second week. The PPIs were to be taken for four weeks. All patients underwent the same treatment protocol and completed it in full.

Clinical outcomes

Clinical follow-up and surveillance testing were performed two weeks after the discontinuation of the PPIs and four weeks after completing the antibiotic treatment. This was done using a monoclonal antigen stool test or the urea breath test. All of our patients underwent these tests, and 94.92% had a favorable outcome, while 5.08% experienced treatment failure, likely due to antibiotic resistance.

## Discussion

*Helicobacter pylori* is one of the most common chronic infections, with a global prevalence of approximately 50% [7]. This infection is primarily acquired during early childhood, particularly in low-income countries. The prevalence of *H. pylori* varies significantly across different countries [7]; in developing nations, around 50% of children are infected before the age of 10. In our study, the prevalence of *H. pylori* infection in children was estimated at 51% (118 cases). In terms of age, the prevalence of this infection increases with age, with the highest affected group being children aged 10 to 16 years, accounting for 46.61%. These results are consistent with a Tunisian study [8], where the most affected age group was also 10 years and older, with a prevalence of 57%.

The exact mode of transmission remains unclear. However, the most likely transmission pathways are gastro-oral via vomit and/or fecal-oral routes. While neither oral nor fecal exposure has been definitively proven as a transmission route, the increased infection rate in children compared to adults supports this hypothesis [9]. Various factors contribute to an elevated risk of *H. pylori* infection, such as poor hygiene practices, a high household population, maternal infection with *H. pylori*, cohabitation in shared living spaces or beds, consumption of untreated or non-boiled water, low socioeconomic status, and inadequate sanitation conditions [7]. These findings are consistent with our study, where 68% of our patients (n=80) had a low socio-economic status, thereby contributing to a higher prevalence of the infection.

*Helicobacter pylori *colonization is a primary cause of chronic gastritis and can lead to more severe conditions, including gastric ulcers, gastric adenocarcinoma, and mucosa-associated lymphoid tissue (MALT) lymphoma in adults. Infection typically occurs in childhood, and the immune responses, both mucosal and humoral, during this period may significantly influence the progression of the infection. Thus, it is crucial to investigate *H. pylori* infection during childhood to better understand its long-term effects [10]. Most individuals infected with *H. pylori* remain asymptomatic and are considered healthy carriers of the bacterium [11]. Current evidence does not establish a definitive causal link between *H. pylori* infection and conditions such as chronic or recurrent abdominal pain or irritable bowel syndrome. Beyond gastrointestinal disorders, *H. pylori* has been associated with systemic conditions, including iron-deficiency anemia and chronic primary immune thrombocytopenia [12]. The clinical symptoms identified in our study align with findings from previous research, such as the Jordanian study by Khdair Ahmed et al. [13], reinforcing that abdominal pain is the most prevalent symptom in children infected with *H. pylori*. Acute gastritis is a broad clinical entity encompassing various conditions that provoke inflammatory alterations in the gastric mucosa. This inflammation arises due to an imbalance between mucosal protective mechanisms and injurious factors, potentially leading to different degrees of gastritis and mucosal ulceration. In this study, *H. pylori *infection was identified through histological analysis using Giemsa staining, a widely utilized technique owing to its affordability, simplicity, and high reproducibility [14]. Micronodular gastritis represents the most distinctive endoscopic feature of *H. pylori* infection in pediatric patients. It is characterized by the presence of micronodules that confer a cobblestone-like appearance to the mucosa. This condition has been reported in 48% to 90% of infected children, in contrast to an incidence of 14.2% in infected adults [15]. In our series, the nodular appearance was observed in 50% of cases, with petechial and erosive features seen in 59% and 62% of cases, respectively. Similarly, an Egyptian study documented both petechial and nodular gastritis, with endoscopic findings ranging from normal mucosal appearance to erythematous lesions [16].

In children, guidelines recommend that the diagnosis of *H. pylori* infection be based on a positive culture or histopathological evidence of gastritis with *H. pylori*, along with at least one other positive biopsy-based test. Once diagnosed, *H. pylori* infection should be eradicated. However, the effectiveness of the standard triple therapy regimen, which includes antibiotics such as amoxicillin, clarithromycin, and metronidazole, along with a PPI, seems to be diminishing due to the emergence of *H. pylori *strains resistant to these treatments. Regarding the epidemiological aspects of treatment, results from the EuroPedHp registry showed that primary antibiotic resistance rates can vary significantly between geographic regions and may also be correlated with migrant status [15].

The eradication of *H. pylori* infection is generally defined as the complete elimination of the bacteria, confirmed by at least two reliable methods (such as the urea breath test and stool antigen test) four to six weeks after the completion of treatment. Tests performed before the completion of four weeks may yield false negative results due to a reduction in bacterial load without complete eradication [17]. In our study, the eradication rate was 94.92%, slightly higher than that observed in other studies, such as the one conducted in Egypt by Metwally et al. [18], where the eradication rate was approximately 90%. This difference may be attributed to the quality of treatment adherence and antibiotic resistance [18].

Although the present study provides valuable information, certain limitations must be acknowledged. The cross-sectional design and limited sample size constrain the ability to thoroughly evaluate the prevalence and risk factors of *H. pylori* infection in the pediatric population of the Eastern region of Morocco. Additionally, the study predominantly focused on symptomatic patients, which may not be fully representative of the entire pediatric cohort, including asymptomatic cases.

## Conclusions

*Helicobacter pylori* remains a significant health concern in children, with clinical presentations and endoscopic findings varying according to age and socioeconomic status. Given its potential to cause long-term gastrointestinal complications, early detection and appropriate treatment are essential to improving clinical outcomes and preventing severe complications such as peptic ulcers and gastritis. In this study, we characterized *H. pylori* infection in Moroccan children from the Eastern region, emphasizing the importance of integrating clinical, endoscopic, and histopathological assessments for a comprehensive evaluation of gastric mucosal health. Furthermore, the strong association between *H. pylori* prevalence and socioeconomic conditions underscores the necessity of implementing widespread prevention and treatment programs. Public health initiatives focusing on improving hygiene, promoting awareness, and ensuring access to appropriate medical care will be crucial in reducing the burden of this infection in pediatric populations. Future research should also explore antibiotic resistance patterns and long-term treatment outcomes to optimize management strategies for affected children.
